# Astaxanthin Restrains Nitrative-Oxidative Peroxidation in Mitochondrial-Mimetic Liposomes: A Pre-Apoptosis Model

**DOI:** 10.3390/md16040126

**Published:** 2018-04-12

**Authors:** Camila M. Mano, Thais Guaratini, Karina H. M. Cardozo, Pio Colepicolo, Etelvino J. H. Bechara, Marcelo P. Barros

**Affiliations:** 1 Departamento de Bioquímica, Instituto de Química, Universidade de São Paulo (IQUSP), 05508-000 São Paulo, SP, Brazil; camilammano@gmail.com (C.M.M.); thais@lychnoflora.com.br (T.G.); karina.cardozo@grupofleury.com.br (K.H.M.C.); piocolep@iq.usp.br (P.C.); 2 Instituto de Ciências da Atividade Física e do Esporte (ICAFE), Universidade Cruzeiro do Sul, 01506-000 São Paulo, SP, Brazil; 3 Superintendência da Polícia Técnico Científica, 05507-060 São Paulo, SP, Brazil; 4 Lychnoflora Pesquisa e Desenvolvimento em Produtos Naturais LTDA, 14030-090 Ribeirão Preto, SP, Brazil; 5 Grupo Fleury, 04344-070 São Paulo, SP, Brazil; 6 Departamento de Ciências Exatas e da Terra, Universidade Federal de São Paulo, Diadema, UNIFESP, 09972-270 Diadema, SP, Brazil; ejhbechara@gmail.com; 7 Departamento de Química Fundamental, Instituto de Química, Universidade de São Paulo (IQUSP), 05508-000 São Paulo, SP, Brazil; 8 Instituto de Agroquímica y Tecnología de Alimentos (IATA-CSIC), Departamento de Ciencia de los Alimentos, Calle Catedrático Agustín Escardino 7, 46980 Paterna, Spain

**Keywords:** mitochondria, membrane, lipid peroxidation, apoptosis, carotenoid, antioxidant, liposome, oxidative stress

## Abstract

Astaxanthin (ASTA) is a ketocarotenoid found in many marine organisms and that affords many benefits to human health. ASTA is particularly effective against radical-mediated lipid peroxidation, and recent findings hypothesize a “mitochondrial-targeted” action of ASTA in cells. Therefore, we examined the protective effects of ASTA against lipid peroxidation in zwitterionic phosphatidylcholine liposomes (PCLs) and anionic phosphatidylcholine: phosphatidylglycerol liposomes (PCPGLs), at different pHs (6.2 to 8.0), which were challenged by oxidizing/nitrating conditions that mimic the regular and preapoptotic redox environment of active mitochondria. Pre-apoptotic conditions were created by oxidized/nitr(osyl)ated cytochrome c and resulted in the highest levels of lipoperoxidation in both PCL and PCPGLs (pH 7.4). ASTA was less protective at acidic conditions, especially in anionic PCPGLs. Our data demonstrated the ability of ASTA to hamper oxidative and nitrative events that lead to cytochrome c-peroxidase apoptosis and lipid peroxidation, although its efficiency changes with pH and lipid composition of membranes.

## 1. Introduction

Dietary carotenoids are thought to provide health benefits by decreasing the risk of disease, particularly those in which free radical metabolism is a central mechanism of disease pathology. Accordingly, many carotenoids are powerful scavengers of reactive oxygen/nitrogen species (ROS/RNS) as well as quenchers of singlet oxygen, a highly reactive species towards proteins, lipids and nucleic acids [1]. Nonetheless, the redox chemistry of carotenoids is very complex at cellular loci and can drastically change under diverse microenvironmental conditions such as dielectric constant, polarity, pH, and temperature. In fact, even prooxidant properties of carotenoids have been described under certain circumstances [2].

Astaxanthin (ASTA) is an orange-pink carotenoid abundant in marine organisms, including algae, zooplankton, mollusks, crustaceans, and salmon [3]. ASTA reportedly exhibits powerful in vitro and in vivo antioxidant and anti-inflammatory activities [4]. Regular or supplementary intake of ASTA in the diet has been shown to afford protection against some free radical-mediated disorders, such as ultraviolet radiation (UV)-promoted skin lesions and cancer, stomach ulcers caused by *Helicobacter pylori* infection, hepatocellular carcinoma, heart failure, macular degeneration, and prostate diseases [5].

Interestingly, a bulk of data supports a plausible mitochondrial-centered action of ASTA in isolated cells, tissues, and even living organisms, although an unequivocal mitochondria-targeted protective mechanism for ASTA has never been shown [6]. Our previous findings demonstrated that ASTA diminishes Ca^2+^ flow, as well as NO^•^, O_2_^•−^, and H_2_O_2_ production in isolated neutrophils and lymphocytes, which were then depicted as putative anti-apoptotic effects [7]. However, the current knowledge of the ASTA-apoptosis relationship is still unclear due to the use of different ASTA concentrations, cell types, and presence of other antioxidants or anti-inflammatory agents [8,9].

Concerning apoptotic mechanisms, oxidative/nitrative modifications of cytochrome c (cyt c) have been suggested as central molecular events underling the activation of caspase pathways in many cells undergoing apoptosis [10]. Peroxynitrite (ONOO^−^) is a powerful oxidizing/nitrating and nucleophilic agent that is generated by the diffusion-limited combination of NO^•^ and O_2_^•−^ radicals in cell. It is also a potent metabolite that modifies the structure and properties of cyt c, thereby triggering apoptosis [11]. In addition to cardiolipin, phospholipids like phosphatidylglycerol (PG) can also change the affinity of both the native- and ONOO^−^-modified forms of cyt c in the inner mitochondrial membrane, thus contributing to the initiation of apoptosis [12].

As regular micronutrients in the human diet, carotenoids deserve more attention to clarify their complex chemistry in biological membranes, especially under different redox and pH conditions. To determine whether the antioxidant properties of ASTA are efficiently sustained under distinct redox conditions that mimic the mitochondrial environment, we exposed unilamellar zwitterionic egg yolk phosphatidyl choline (PCLs) and anionic phosphatidyl choline/phosphatidyl glycerol liposomes (PCPGLs) to oxidative/nitrative lesions that normally occur in mitochondria. In addition, an apoptosis-like condition was imposed by oxidized/nitr(osyl)ated cyt c at pHs ranging from 6.2 to 8.0. Our working hypothesis is that ASTA displays antioxidant properties that are modulated by the pH and lipid composition of membranes due to the structural effects of ASTA in terms of membrane fluidity/dynamics and/or to the association of cyt c (modified or not) with lipid bilayers under such conditions.

## 2. Results

### 2.1. Oxidative/Nitrative Modifications on Cytochrome C

The thermal decomposition of 3-morpholinosydnonimine (SIN-1) releases “free” superoxide (O_2_^•−^) and nitric oxide (NO^•^) radicals in air-equilibrated aqueous solution during a two-step process that uses the cationic sydnonimine intermediate SIN-1A^•+^ and yields (4-morpholinylimino) acetonitrile (SIN-1C) as the final product (Figure S1A). None of these compounds absorbs visible light near the typical cyt c Soret band at λ_max_ = 409 nm, a wavelength peak that defines the redox behavior of cyt c (Figure S1B).

**Figure 1:**
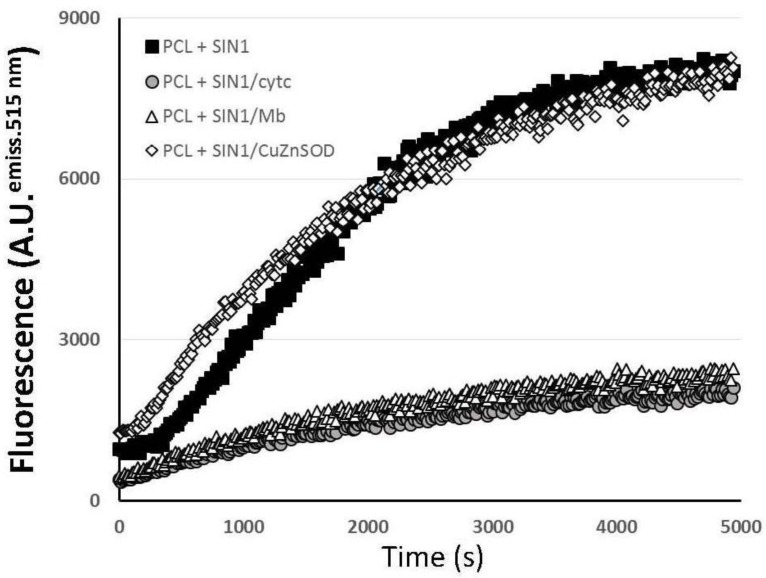
Fluorescence of 4-amino-5-methylamino-2′,7′-difluorofluorescein (DAF-FM) with 3-morpholinosydnonimine (SIN-1) decomposition in 50 mM phosphate buffer, pH 7.4, containing egg yolk phosphatidyl choline liposomes (PCL), hemeproteins (cytochrome c; cytc, or myoglobin; Mb) or Cu,Zn-dependent superoxide dismutase (CuZnSOD).

Free NO^•^ in aqueous solution was identified by the fluorescent probe 4-amino-5-methylamino-2′,7′-difluorofluorescein (DAF-FM) in PCL at pH 7.4 (Figure 1). A similar DAF-FM kinetic pattern was observed in 10% PCPGLs (Figure S2). The kinetics of NO^•^ release was substantially hindered by the presence of both hemeproteins, cyt c and, for comparison, myoglobin (Mb). Addition of superoxide dismutase (SOD), which totally consumes the O_2_^•−^ radical, did not affect the identification of the NO^•^ signal in aqueous solution.

Supplementary material 3 (Figure S3) shows the 150-µM cyt c UV-Vis spectrum after a 45-min treatment with 150 µM *N*-[4-[1-(3-aminopropyl)-2-hydroxy-2-nitrosohydrazino] butyl-1,3-propanediamine (spermine NONOate), an exclusive NO^•^ donor, in aerated aqueous solution at pH 7.4. The coordination of NO^•^ by the heme iron of ferricytochrome c can be observed by the appearance of a peak at 562 nm. Expectedly, no changes were observed in the Soret band at 409 nm [13].

**Figure 2:**
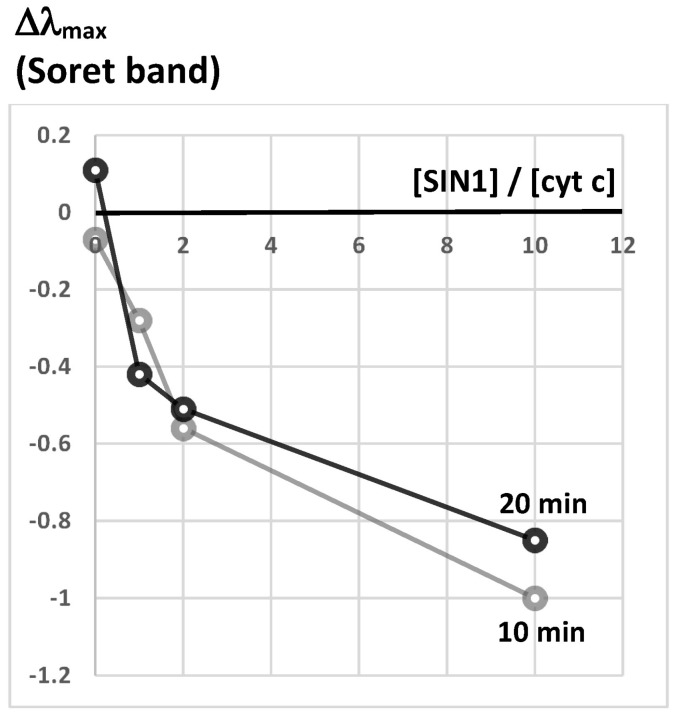
Shift of the cyt c Soret band (Δλ_max_) treated with SIN-1 with PCL, in 50 mM phosphate buffer, pH 7.4, for 20 min.

To detect structural heme pocket distortions induced by SIN-1 in cyt c (Soret band at 409 nm), the absorbance was scanned at 10 and 20 min reaction times from 370 to 450 nm. The calculated parameters of the spectrum smoothing procedure [fitting the Gaussian function, Equation (1)] are presented in Supplementary Materials 4 (Table S1), including Soret λ_max_ (x_c_ parameter). Figure 2 summarizes the observed blue shift of the calculated cyt c Soret band at different SIN-1/cyt c ratios mixed with PCL in 50 mM phosphate buffer, pH 7.4, which indicates the permanent acquisition of a peroxidase-like activity).

### 2.2. Peroxidation of Liposomes by the SIN-1/Cyt C System

**Figure 3:**
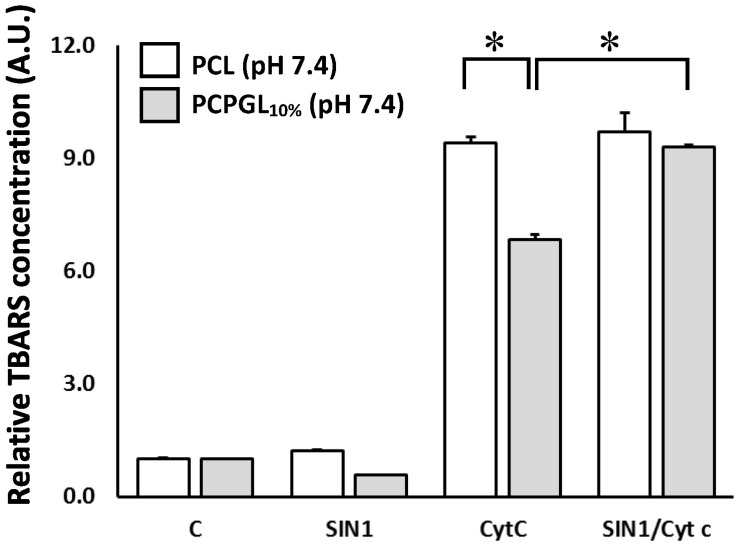
Thiobarbituric acid-reactive substances (TBARS) levels of PCL or phosphatidyl choline:phosphatidyl glycerol liposomes (PCPGLs) treated with cyt c, and/or SIN-1, at pH 7.4. (* *p* ≤ 0.05).

In comparison with untreated liposomes (control, C), both cyt c and SIN-1/cyt c resulted in a nine-fold increase in lipoperoxidation, expressed as the concentration of thiobarbituric acid-reactive substances (TBARS) in PCL (Figure 3). Similar increase was also observed when 10% PCPGL was treated with SIN-1/cyt c, however lipid peroxidation was reduced by 25% when cyt c alone was used to oxidize these liposomes.

### 2.3. Astaxanthin Effect on Liposome Size

As shown in Table 1, all liposomes showed equal diameters within experimental error both in the presence and absence of ASTA (Dynamic Light Scattering data).

### 2.4. pH Effect on Lipid Peroxidation and Astaxanthin Antioxidant Properties

**Figure 4:**
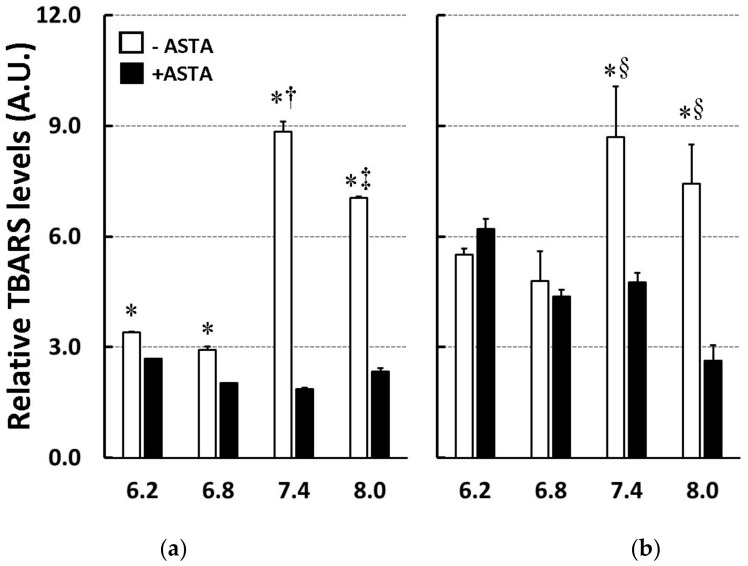
TBARS levels of (**a**) PCL or (**b**) PCPGLs, loaded (+ASTA) or not (-ASTA) with 25 µM Astaxanthin (ASTA) and treated with SIN-1/cyt.c in 50 mM phosphate buffer, in different pH. * *p* ≤ 0.05 compared to respective ASTA-loaded liposome; § *p* ≤ 0.05 compared to pH 6.2 and 6.8; † *p* ≤ 0.05 compared to pH 6.2, 6.8, and 8.0; and ‡ *p* ≤ 0.05 compared to 6.2, 6.8, and 7.4.

Thiobarbituric acid-reactive substances levels were also measured in PCL and 10% PCPGLs oxidized by SIN-1/cyt c in 50 mM phosphate buffer at different pHs. Using non-oxidized liposomes as a reference (control in Table 2), the data depicted by Figure 4 show that lipid peroxidation of PCL was lower in acidic conditions compared to pHs 7.4 and 8.0. These oxidative conditions were counteracted by 25 µM ASTA, which provided maximal protection to membranes at pH 7.4 (80%), whereas TBARS inhibition at pH 8.0 was around 65%. ASTA slightly inhibited lipid peroxidation in PCL at pHs 6.2 (–20%) and 6.8 (–30%). In anionic PCPGLs, TBARS levels at pHs 6.2 and 6.8 were almost two-fold higher than in PCL. No differences were observed between TBARS levels in PCL and 10% PCPGLs at pHs 7.4 and 8.0. In summary, ASTA exhibited no antioxidant effects in 10% PCPGLs at acidic conditions, but only at pHs 7.4 (–44%) and 8.0 (–65%), offering less antioxidant protection than that observed in PCL at the same pHs. The effect of ASTA against lipoperoxidation induced by other mitochondrial-mimicking oxidative/nitrative conditions in PCL and 10% PCPGL (see Materials and Methods, Section 4.5. Oxidative/Nitrative Conditions for Liposome Peroxidation), is presented in Table 2.

**Figure 5:**
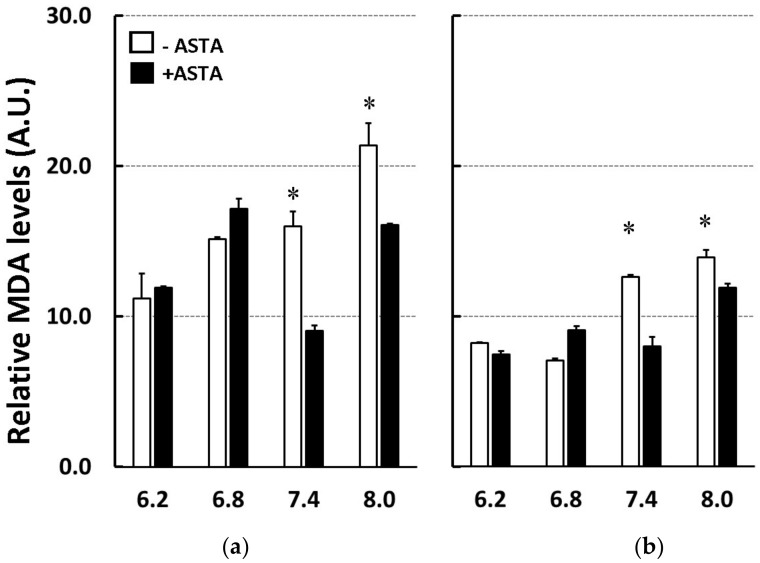
MDA levels of (**a**) PCL or (**b**) PCPGLs treated with SIN-1/cyt.c, in the presence or absence of 25 µM ASTA, in 50 mM phosphate buffer, in different pH. * *p* ≤ 0.05.

The peroxidation data monitored by malondialdehyde (MDA) concentrations in PCL and 10% PCPGLs (Figure 5) were comparatively similar to those observed through the TBARS assay in Figure 4. Accordingly, 25 µM ASTA did not show any significant inhibition of lipid peroxidation measured as MDA levels at acidic conditions in either PCL or 10% PCPGLs. Significant antioxidant activity of ASTA was therefore maximal at pH 7.4 in both PCL (–45%) and 10% PCPGLs (–37%). At pH 8.0, ASTA inhibited MDA-lipid peroxidation by 24% in PCL and 15% in 10% PCPGLs (Figure 5). The effect of 25 µM ASTA against MDA-HPLC formation induced by other mitochondrial-mimicking oxidative/nitrative conditions in PCL and 10% PCPGL (Section 4.5. Oxidative/Nitrative Conditions for Liposome Peroxidation), is presented in Table 3.

## 3. Discussion

### 3.1. Cytochrome c-Modified by SIN-1 Induces Liposome Peroxidation

We aimed here to unveil the antioxidant properties of ASTA in a redox environment similar to that found in active mitochondria, including that linked to the initiation of apoptosis via cyt c oxidation/nitr(osyl)ation [14]. The combination of ROS/RNS such as O_2_^•−^, NO^•^, and ONOO^−^ is known to change the redox behavior of cyt c in mitochondria, specifically its peroxidase activity and increased permeability via high valence heme intermediates [11]. Importantly, recent evidence shows that ASTA possibly targets the mitochondria in many animal cell models under a wide variety of physiological conditions [15].

First, redox alterations of cyt c upon treatment with SIN-1, a synthetic compound that decomposes into O_2_^•−^ and NO^•^ in aqueous solution [16] was revisited at planned experimental conditions. Our main findings are as follows: (i) NO^•^ formed from SIN-1 decomposition interacts with heme group of cyt c (as well as myoglobin, for comparison) independently upon the presence of O_2_^•−^ (Figure 1 and Figure S2); (ii) structural distortions in the vicinity of the heme group of cyt c are induced by SIN-1 treatment (Table S4 and Figure 2), which brings about redox changes of the protein properties (Figure 3); (iii) the cyt c misshapes were not caused by NO^•^ alone (Figure S3) as suggested by the lack of effect in the presence of spermidine NONOate; instead, protein changes were only observed in the presence of SIN-1-formed O_2_^•−^ and NO^•^; (iv) SIN-1 treatment of cyt c triggers the highest oxidizing conditions for all liposome systems (Figure 3); and (v) most importantly, the extent of lipid peroxidation is roughly identical in zwitterionic PCL or anionic 10% PCPGLs after SIN-1/cyt c treatment (Figure 3). Although extensively studied, the mechanism of cyt c-catalyzed peroxidation of membrane phospholipids is not yet well understood [17]. Previous work has shown that ONOO^−^ is not likely to drive cyt c modifications under the experimental conditions used here, since SIN-1-treated cyt c does not exhibit the spectral characteristics of nitrated cyt c produced by direct reaction with ONOO^−^ [18].

The TBARS data shown in Figure 3 reveal that cyt c alone can catalyze lipid peroxidation in zwitterionic PCL at pH 7.4. Additional treatment with SIN-1 does not exacerbate the cyt c-induced lipid peroxidation process induced in these PCL, suggesting that SIN-1 does not fully or concomitantly interact with cyt c. Or else, it indicates that SIN-1-derived ROS/RNS does not impose further structural distortions in cyt c. Interestingly, TBARS levels were 35% lower in 10% PCPGLs compared to PCL treated with cyt c alone (Figure 3). It is expected that the cationic heme protein cyt c, (pI ~ 10.4, pH 7.4) has stronger electrostatic interactions with anionic membranes of PCPGLs. Nevertheless, such protein-lipid interactions were apparently insufficient to initiate lipid peroxidation compared to that observed in PCL. The presence of water-soluble lipid hydroperoxides formed during the preparation of liposomes could also possibly initiate the cyt c catalytic cycle under our experimental conditions here. Further experiments are necessary to investigate this hypothesis.

### 3.2. Liposome Size/Organization is Unaltered by Astaxanthin Incorporation

Liposome sizes were not affected by lipid composition or the association of ASTA with PCL or 10% PCPGLs (Table 1). Available data show that the incorporation of phosphatidylglycerol (PG) into PCL (of different compositions) does not affect liposome diameter/size, within the pH range studied here [19].

With respect to accessibility of ROS/RNS in liposomal membranes, the unsaturation index of phosphatidylcholine (PC) or PG was shown to influence the distance across phospholipid head groups [20]. Although the lipid composition was not actually measured in either PC or PG in this study, we can assume, based on the manufacturer’s description (Sigma-Aldrich, St. Louis, MO, USA), that the unsaturation content is similar in both phospholipids, implying that both liposomal systems have an equivalent number of targets and identical accessibility of ROS/RNS for lipid peroxidation. Finally, Xia et al. (2015) also showed that only concentrations above 1% xanthophyll (canthaxanthin), with similar polar features as ASTA, diminish membrane fluidity (with head group region reorganization) in egg yolk PCL, compared to β-carotene and lutein [21]. The effective ASTA concentration used here was 25 µM, corresponding to 0.5% of total phospholipids in membranes.

In summary, observable differences in lipid peroxidation at different pHs could not be attributed to compacting effects on the head groups (becoming less accessible to pro-oxidants) or to significant changes in the water-lipid contact surface (an effect of changes in size or diameter) related to different phospholipid compositions or ASTA associations in our experimental liposome preparations.

### 3.3. Astaxanthin Suppresses Cyt C/SIN-1-Induced Liposome Oxidation at pH > 7.4

Active mitochondria sustain a high membrane potential (ΔΨ and ΔpH during regular aerobic metabolism. Performing calibrations with the MitoTracker Red probe (Molecular Probes, Eugene, OR, USA), Porcelli et al. [22] estimated the pHs in the intermembrane space, cytosol, and mitochondrial matrix to be 6.88 ± 0.09, 7.59 ± 0.01, and 7.78 ± 0.09, respectively. On the other hand, key apoptotic events such as cyt c-activated caspase cascades and apoptotic Bcl-2/Bax pore formation involve the mild acidification of the cytosol down to 0.5 pH units [23]. The activation of caspase pathway by native cyt c is optimum at pH 6.4, but only 25% at pH 7.4 [24]. In the present work, the pH range scrutinized (6.2 to 8.0) encompasses the hydrogen ion concentrations reported in active mitochondrial compartments during either regular metabolism or apoptosis.

Within the lipid bilayer, the intrinsic pKa of the phosphate group in PC is 0.8 as determined in monolayers using an Hg-drop adsorption electrode [25]. The lack of the choline tetrammonium group moiety, turns PG more anionic than PC at pH 7.4, although no abrupt changes in the phosphate group pKa are expected to occur. Considering that the pKa of PC is 0.8, no significant net charge variations in either PCL or 10% PCPGLs would be observed in the experimental pH range studied here. The most significant effect of pH on lipid peroxidation is long known to occur in the presence of catalytic ferrous/ferric ions due to the production of hydroxyl radicals (HO^•^) involving Fe(OH)_2_ and Fe(OH)_3_ interconversion (Fenton reaction).

The pH is crucial to attain effective oxidizing/nitr(osyl)ating conditions to trigger peroxidation of lipid systems. Although SIN-1 decomposition generates O_2_^•−^ and NO^•^ radicals which decay to the oxidizing/nitrifying agent ONOO^−^ [26], the latter species does not seem to be directly involved in cyt c modifications under our experimental conditions. Although iron-induced lipid peroxidation is enhanced at decreasing extracellular pH values [27], our data show that both TBARS and MDA levels were diminished at pHs 6.2 and 6.8 as compared to pHs 7.4 and 8.0 (Figure 3 and Figure 4). This finding suggests a role for “free” ferrous ions in the initiation of lipid peroxidation upon SIN-1/cyt c treatment. Conformational changes surrounding the Met_80_ ligand (sixth coordination site) of cyt c and its replacement by a Lys residue (probably Lys_79_) has an apparent pKa of approximately 9.0 [17]. Therefore, one can assume that no more than a 10% increase in cyt c-peroxidase activity would be expected from a pH increase within the pH 6.2–8.0 range.

In summary, the pH range studied here is in accordance with the proton concentration in active mitochondrial compartments, thus favoring the initiation of apoptosis. Higher pH does not significantly enhance the peroxidase activation of cyt c or induce net membrane charges in either PCL (zwitterionic) or 10% PCPGLs (anionic). Despite refuting the participation of peroxynitrite from SIN-1 decomposition here, the same array of ROS/RNS subsequently induce lipid peroxidation of PCL and 10% PCPGL. Thus, one can assume that any pH-dependent changes in lipid peroxidation in liposomal systems would be solely caused by the varying scavenging properties of ASTA within membranes, for ASTA’s effects on membrane physicochemical properties are also not plausible.

### 3.4. Mechanism of Astaxanthin-Protective Role against Liposome Peroxidation

ASTA usually achieves higher thermodynamic stability in lipid bilayers by allowing its carboxyl and hydroxyl polar groups to interact with the hydrophilic head groups of phospholipids on the opposite sides of the membrane, thus assuming a ‘vertical’ orientation across the lipid bilayer [28]. However, at a concentration of 25 µM (0.5% compared to total lipid content in both PCL and PCPGL), no significant structural modifications induced by the association of ASTA with liposomes would cause significant changes in membrane fluidity and so effectively altering membrane permeabilization for water-soluble, pro-oxidative agents such as H_2_O_2_ and NO^•^.

Noteworthy is that El-Agamey et al [29] used pulse radiolysis in an aqueous, 2% argon-saturated (*v/v*) Triton X-100 solution to study the effects of pH on ASTA anion radicals (ASTA^•−^) derived from the scavenging activity of ASTA [29]. The pKa value of the ASTAH^•^/ASTA^•−^ pair was found to be 10.62 in a micelle-aqueous solution. This suggests that no significant fraction of ASTA^•−^ is expected at pH 6.2 (<0.004%) or pH 8.0 (<0.3%). Considering the pKa value of 10.62 and, thereby, the predominant protonated form of ASTAH^•^ radical, we can exclude the hypothesis that electrostatic repulsions of ASTA^•−^ radicals within membranes could interfere with the propagation and termination steps of lipid peroxidation in PC or 10% PCPGLs.

*Cis,trans*-isomerization of the ASTA polyene chain is also a pH-dependent feature of carotenoid chemistry. Liu & Osawa (2007) reported that 9-*cis*-ASTA exhibits higher antioxidant activity than the all-trans isomer in vitro [30]. However, while the fraction of 9-*cis*-ASTA isomers increases under heat and acidic conditions [31], ASTA displayed less inhibitory activity toward lipid peroxidation under more acidic conditions (pHs 6.2 and 6.8), as measured by either TBARS or MDA levels. Nevertheless, the percentage of *cis-trans* interconversion cannot explain the substantial antioxidant protection that ASTA elicits in PCL or PCPGLs from 37% to 45%, at different pHs.

Previous work has shown that ASTA preferentially blocks lipid peroxidation at the polar membrane surface of egg yolk PC liposomes [28]. ASTA directly intercepts O_2_^•−^, NO_2_^•^, and HO^•^ radicals, produced from SIN-1 decomposition in addition to lipoperoxyl (LOO^•^) and lipoxyl (LO^•^) radicals formed during propagation of lipid peroxidation. Another relevant feature of ASTA chemistry is the formation of hydrogen bonds involving C=O and -OH groups attached to its β-ionone rings. Intermolecular hydrogen bonds are normally formed between the two terminal polar rings of ASTA (hydroxyl-keto groups) and the polar phospholipid head groups, but there is also evidence that ASTA forms intramolecular hydrogen bonds between those same polar head groups [28]. The formation of intramolecular hydrogen-bonded five-membered rings increase the hydrophobicity of ASTA and favor its insertion into the hydrophobic core of the lipid bilayer. Such a translocation of the terminal rings of ASTA should be advantageous for scavenging lipid-soluble radicals such as LOO^•^ and LO^•^. In fact, ASTA is able to trap radicals by its conjugated polyene chain at the hydrophobic core of membranes, and in both hydrophobic core and water-lipid interfaces by its terminal ring moiety, based on switches between intramolecular hydrogen bonds and those formed with the polar groups of phospholipids [28]. Another hypothesis is that pH could influence the water-lipid surface/hydrophobic core distribution of ASTA molecules by keto-enol tautomerism. As an enolate, a negatively charged molecule, ASTA could not insert into the hydrophobic core of membranes to perform its scavenging properties. The calculation of the pKa of the enol/enolate form of ASTA is approximately 9.5 [32].

Therefore, among all the pH-dependent factors investigated here, tautomerism of the ASTA keto-enol forms, pH-dependent dissociation to its enolate form, and the formation of hydrogen bonds (intramolecular or with polar groups of phospholipids) are apparently the most plausible aspects of the complex chemistry of ASTA that could explain its varying antioxidant activity against mitochondrial-like redox conditions.

## 4. Materials and Methods

### 4.1. Chemicals

All chemicals used were obtained from Sigma-Aldrich Brasil Ltda. (São Paulo, Brazil) and the liquid chromatography grade solvents—n-hexane, chloroform, methanol and ethanol—purchased from Merck Co. (Darmstadt, Germany).

### 4.2. Astaxanthin Stock Solutions

Stock solutions of ASTA (10 mM) were prepared in chloroform, of which the concentrations were determined by spectrophotometry (ε = 101 L·mol^−1^.cm^−1^ at 486 nm). Stock solutions were stored at −80 °C and protected from light to avoid oxidation.

### 4.3. Preparation of Unilamellar Liposomes

Egg-yolk PC and egg-yolk PG were selected to prepare liposomes once: (i) PC and PG abound in biological membranes; (ii) egg-yolk phospholipids are polyunsaturated fatty acids (PUFAs)-rich, which are prone to suffer oxidative damage; and (iii) PC and PG have distinct net charges under different pH conditions thereby able to affect protein-lipid interactions in liposome systems. Astaxanthin stock solutions were previously mixed with phospholipid solutions in chloroform to reach a final carotenoid:phospholipid ratio of 0.5% mol ASTA, in order to avoid carotenoid aggregation in the formed liposomes. Final concentration of ASTA in liposomes was 25 µM. Control liposomes lack ASTA. Three liposome systems were prepared:

- 5 mM phosphatidylcholine (5 mM PCL);
- 4.76 mM phosphatidylcholine + 0.24 mM phpsphatidylglycerol (PCPGL 5%); and
- 4.54 mM phosphatidylcholine + 0.46 mM phpsphatidylglycerol (PCPGL 10%).

Chloroform was subsequently removed by flushing N_2_ in a round-bottom flask adapted to a rotavapor apparatus working at a low speed and moderate heating (≤37 °C) to allow the formation of a homogeneous dried ASTA: phospholipid film. The lipid-carotenoid film was stored overnight in the dark under vacuum to eliminate traces of solvent.

Multilamellar liposomes were prepared by vortexing volumes of 50 mM phosphate buffer (pH 6.2, 6.8, 7.4, or 8.0) added to the lipid film for 5 min, at room temperature. Finally, unilamellar liposomes were prepared by extrusion through 100 µm-pore polycarbonate membranes (MilliPore, Burlington, MA, USA), at 37 °C, in a mini-extruder device (Avanti Polar Lipids, Inc., Alabaster, AL, USA). Through 15 passes, we obtained clean, transparent and homogeneous unilamellar PCL or PCPGL suspensions and immediately used them in the experiments. Usual sonication was avoided to prepare our liposomal systems here, since it precludes the release of Ti^3+^ ions into the reaction milieu (from the sonicator tip), which would artifactually induce lipid oxidation in liposomes during procedures.

### 4.4. Dynamic Light Scattering Studies

Dynamic light scattering (DLS) was used to determine the average liposome size in a Malvern Zetasizer Nano-series equipment (Malvern Instruments Zen 600, Malvern, UK). Aliquots from PC, PCPG5% e PCPG10% (with or without ASTA) stock solutions were diluted to a final concentration of 1 mM total phospholipid in 50 mM phosphate buffer, pH 7.4, at 25 °C. To check ASTA incorporation into liposome membranes laser with λ = 590 nm was used.

### 4.5. Oxidative/Nitrative Conditions for Liposome Peroxidation

Five oxidative/nitrative different conditions (plus control) were studied to initiate lipid peroxidation in the normally aerated liposomal systems, in an attempt to mimic the regular redox environment in mitochondria. A 150 µM 3-morpholinosydnonimine (SIN-1) solution was used as a biphasic generator of O_2_^•−^ and NO^•^ radicals, and presumably peroxynitrite (ONOO^−^ ) as the O_2_^•−^/ NO^•^ annihilation second order constant is ~10^10^ M^−1^ s^−1^. Spermin NONOate (150 µM) was used as a single NO^•^ donor. Based on previous studies, an equimolar (150 µM) mixture of SIN-1 and cyt c better reproduces the redox scenario of the mitochondrial apoptotic condition [16]. All experiments and control tests were performed in 50 mM phosphate buffer at various pHs, 37 °C, for 82 min, considering the SIN-1 decay kinetics with t_1/2_ = 2900 s. The oxidative/nitrative conditions (final concentrations in 1 mL) are presented below:

- 5 mM PCL/PCPGL + 50 mM tPi (pH 6.2–8);
- 5 mM PCL/PCPGL + 50 mM tPi (pH 6.2–8) + 150 µM SIN-1;
- 5 mM PCL/PCPGL + 50 mM tPi (pH 6.2–8) + 150 µM SIN-1 + 150 µM cyt c;
- 5 mM PCL/PCPGL + 50 mM tPi (pH 6.2–8) + 150 µM KO_2_;
- 5 mM PCL/PCPGL + 50 mM tPi (pH 6.2–8) + 150 µM SIN-1 + 0.6 U/mL SOD;
- 5 mM PCL/PCPGL + 50 mM tPi (pH 6.2–8) + 150 µM KO_2_ + 0.6 U/mL SOD;

where: tPi = phosphate buffer; KO_2_ = potassium superoxide; and SOD = superoxide dismutase.

### 4.6. UV-Vis Spectrophotometry

Absorption spectra were traced on a Varian Carry 50 Bio UV-VIS spectrophotometer (Agilent Technologies, Santa Clara, CA, USA) at 37 °C. The time course of the thermal decomposition of SIN-1 was followed at 290 nm, and product formation at 275 nm. Structural changes of cyt c during reactions with SIN-1 were accompanied by scanning the 390–430 nm λ_max_ shifts of the cyt c Soret band, which indicates any possible disruption of the hexacoordinate form of the heme iron [33]. Different SIN-1:cyt c ratios were employed to analyze the extent of SIN-1-promoted oxidative/nitrating effects to the hemeprotein: (i) 50 µM cyt c + 0 µM SIN-1 (control); (ii) 5 µM cyt c + 50 µM SIN-1; (iii) 50 µM cyt c + 50 µM SIN-1; and (iv) 50 µM cyt c + 100 µM SIN-1. The spectral background noise, which compromises the detection of slight shifts in the λ_max_ of the Soret band, was taken into account by adjusting the spectral data to a smoothing Gaussian-function (390–430 nm), defined by the formula:

y = y_0_ + [A/(w(π/2)^1/2^)] e^−2.[(x−xc)2/w2]^), (1)

where: A, is the area under curve; w, the band width (data distribution); y_0_, the curve cut off (baseline); R^2^, the correlation index; and xc, the curve peak (calculated λ_max_).

### 4.7. Nitric Oxide Concentration

The NO^•^ concentration was measured by the fluorescence intensity (λ_exc_ = 495 nm; λ_em_ = 515 nm) of the probe 4-amino-5-methylamino-2′,7′-difluorofluorescein (DAF-FM; Molecular Probes^®^, Fisher Scientific, Pittsburgh, PA, USA). The manufacturers state that the fluorescence quantum yield (ϕ_F_) of DAF-FM is c.a. 0.005, which roughly increases 160-fold after reacting with NO^•^ (ϕ_F_ ~ 0.81) in a pH-independent process, pH > 5.5. 

### 4.8. Indices of Lipid Peroxidation

Lipid peroxidation was evaluated from the observed concentrations of thiobarbituric acid-reactive substances (TBARS; spectrophotometric assay) and malondialdehyde (MDA; HPLC-UV/VIS detection) in the spent liposome oxidation sample. The peroxidation reactions were stopped by adding 8 mM butylated hydroxytoluene (BHT) dissolved in ethanol. TBARS detection was performed with 200 µL of sample after incubation at 100 °C for 15 min with 500 µL of 0.25% thiobarbituric acid in 0.25 M HCl, and 2% Triton X-100. After reaching room temperature, absorbances were measured at 535 nm (blanks lack thiobarbituric acid) using 1,1′,2,2′-tetraethoxypropane as a standard, which decomposes in water to form malondialdehyde (MDA) [34]. After cleaning procedures, 20 µL of the solution were injected into a Shimadzu SCL 10A HPLC system provided with a fluorescence detector RF-10AXL (Shimadzu Group Co., Kyoto, Japan) and isocratically eluted through a C_18_-Bondapack 0.39 × 30 cm column by a 65:35 mixture of 25 mM phosphate buffer (pH 6.5) and 30% methanol. The integrated area under MDA peaks (retention time ~ 12 min) was used to calculate MDA concentration based on a standard curve identical to that traced for the TBARS assay [35]. TBARS and MDA values were presented as relative values (A.U.) compared to respective controls in Figure 3, Figure 4 and Figure 5 and Table 2 and Table 3.

### 4.9. Statistical Analysis

Data are presented as mean ± standard deviation, (x ± SD, *n* ≥ 3) and the statistical analysis performed with the Student’s t test at significance level of 5% (OriginPro 2016, 64-bit, Sr-2). Graphics were plotted with Excel 2016, Microsoft Office 365, and OriginPro 2016, 64-bit, Sr-2.

## 5. Conclusions

This work contributes to a better understanding of the complex biochemistry of ASTA related to the scavenging of relevant ROS/RNS under different pHs and lipid membrane compositions. As shown here, ASTA presents a modest antioxidant activity under acidic conditions when zwitterionic or anionic liposomes are peroxidized during our apoptosis-like SIN-1/cyt c model system. Overwhelming efficacy of ASTA was however testified here under physiological (pH 7.4) and mild alkaline conditions (pH 8.0) in both PC and PG liposomal systems. The tautomerism of ASTA keto-enol forms (pH-dependent enolate formation) and the formation of hydrogen bonds (intramolecular or with polar groups of phospholipids) imply different antioxidant activities and thus better explain its antioxidant behavior. Once mitochondrial metabolism involves variations in ΔΨ and ΔpH, we hypothesize that ASTA is endowed with pH-dependent antioxidant/antiapoptotic properties in respiring mitochondria.

## Figures and Tables

**Table 1:** Average diameters of PCLs or PCPGLs in 50 mM phosphate buffer, pH 7.4, with/without 25 µM ASTA (*n* = 3).

Liposome	Control (Diameter, nm)	+25 µM ASTA (Diameter, nm)
PCL	196.7 ± 0.8	184.2 ± 0.6
5% PCPGL	191.5 ± 0.3	181.2 ± 0.3
10% PCPGL	189.1 ± 1.3	190.4 ± 0.4

**Table 2:** TBARS levels of PCL or PCPGLs treated with SIN-1, KO_2_, and/or SOD, in 50 mM phosphate buffer, in different pH (* *p* ≤ 0.05).

TBARS Levels		pH							
		6.2		6.8		7.4		8.0	
		−ASTA	+ASTA	−ASTA	+ASTA	−ASTA	+ASTA	−ASTA	+ASTA
PCL	C	1.00 ± 0.04	1.00 ± 0.04	1.00 ± 0.13	1.00 ± 0.01	1.00 ± 0.08	1.00 ± 0.02	1.00 ± 0.01	1.00 ± 0.05
	SIN1	0.77 ± 0.01	1.19 ± 0.01	0.88 ± 0.02	0.95 ± 0.01	0.82 ± 0.04	1.10 ± 0.02	1.14 ± 0.03	1.02 ± 0.03
	KO_2_	0.79 ± 0.05	1.03 ± 0.03	0.77 ± 0.22	0.92 ± 0.04	1.06 ± 0.07	1.04 ± 0.01	0.98 ± 0.05	0.97 ± 0.02
	SIN1+SOD	0.92 ± 0.01	1.18 ± 0.03	0.90 ± 0.03	0.95 ± 0.07	1.09 ± 0.05	1.15 ± 0.02	1.26 ± 0.03	1.06 ± 0.03
	KO_2_+SOD	0.71 ± 0.01	1.07 ± 0.01	1.00 ± 0.03	0.95 ± 0.06	1.18 ± 0.10	1.06 ± 0.01	0.84 ± 0.04	1.12 ± 0.05
PCPGL 10%	C	1.00 ± 0.61	1.00 ± 0.19	1.00 ± 0.39	1.00 ± 0.46	1.00 ± 0.03	1.00 ± 0.01	1.00 ± 0.03	1.00 ± 0.03
	SIN1	1.56 ± 0.79	1.21 ± 0.20	1.24 ± 0.47	1.35 ± 1.00	1.38 ± 0.01	0.88 ± 0.01	1.14 ± 0.01	0.90 ± 0.04
	KO_2_	2.25 ± 0.49	1.32 ± 0.28	1.16 ± 0.26	1.93 ± 0.49	1.10 ± 0.05	0.95 ± 0.02	0.95 ± 0.04	0.89 ± 0.02
	SIN1+SOD	1.91 ± 0.60	1.11 ± 0.26	1.09 ± 0.65	0.86 ± 0.37	1.53 ± 0.01	0.90 ± 0.03	1.13 ± 0.01	0.92 ± 0.01
	KO_2_+SOD	1.48 ± 0.40	2.21 ± 0.25	1.49 ± 0.47	1.30 ± 0.35	1.29 ± 0.02	0.97 ± 0.02	1.07 ± 0.03	0.92 ± 0.02

**Table 3:** MDA levels of PCL or PCPGLs treated with SIN-1, KO_2_, and/or SOD, in 50 mM phosphate buffer, in different pH (* *p* ≤ 0.05).

MDA-HPLC Levels		pH							
		6.2		6.8		7.4		8.0	
		−ASTA	+ASTA	−ASTA	+ASTA	−ASTA	+ASTA	−ASTA	+ASTA
PCL	C	1.00 ± 0.06	1.47 ± 0.15	1.00 ± 0.16	1.29 ± 0.01	1.00 ± 0.01	1.07 ± 0.01	1.00 ± 0.03	1.69 ± 0.30
	SIN1	1.00 ± 0.03	1.37 ± 0.05	1.37 ± 0.20	1.78 ± 0.06	0.65 ± 0.02	0.84 ± 0.02	1.51 ± 0.01	1.72 ± 0.01
	KO_2_	1.17 ± 0.03	i.d.	1.29 ± 0.30	i.d.	1.28 ± 0.01	1.01 ± 0.01	1.10 ± 0.16	1.63 ± 0.01
	SIN1+SOD	1.13 ± 0.08	1.35 ± 0.07	1.44 ± 0.19	1.70 ± 0.01	0.75 ± 0.01	0.87 ± 0.02	1.92 ± 0.10	1.34 ± 0.01
	KO_2_+SOD	0.95 ± 0.19	i.d.	1.02 ± 0.03	1.92 ± 0.01	1.20 ± 0.03	1.23 ± 0.02	0.99 ± 0.03	1.50 ± 0.21
PCPGL 10%	C	1.00 ± 0.11	1.15 ± 0.10	1.00 ± 0.09	0.99 ± 0.13	1.00 ± 0.08	1.11 ± 0.06	1.00 ± 0.09	1.00 ± 0.08
	SIN1	1.06 ± 0.18	1.27 ± 0.09	1.07 ± 0.09	1.08 ± 0.09	1.09 ± 0.13	1.69 ± 0.06	0.55 ± 0.01	1.35 ± 0.14
	KO_2_	1.06 ± 0.13	1.24 ± 0.26	1.07 ± 0.11	1.01 ± 0.14	1.13 ± 0.14	1.14 ± 0.04	0.97 ± 0.06	1.07 ± 0.02
	SIN1+SOD	1.17 ± 0.18	1.18 ± 0.06	1.08 ± 0.09	1.19 ± 0.08	1.04 ± 0.12	1.00 ± 0.14	1.13 ± 0.01	1.07 ± 0.14
	KO_2_+SOD	1.20 ± 0.05	1.20 ± 0.14	1.24 ± 0.10	1.21 ± 0.15	1.00 ± 0.10	1.02 ± 0.06	1.07 ± 0.17	1.00 ± 0.41

i.d. = inconsistent data.

## Supplementary Materials

The following are available online at http://www.mdpi.com/1660-3397/16/4/126/s1, Figure S1: (a) Decomposition mechanism of SIN-1 in aerated aqueous solution; (b) UV-Vis spectra of SIN-1 decomposition, for 90 min, in 50 mM phosphate buffer, pH 7.4, Figure S2: Fluorescence of the NO^•^ sensitive probe DAF-FM in the presence of PCPGL, in 50 mM phosphate buffer, pH 7.4, Figure S3: UV-Vis spectra of cyt c treated with spermine NONOate. Insert: fluorescence of DAF-FM as a function of the decomposition of spermine NONOate in 50 mM phosphate buffer, pH 7.4, Table S1: Gaussian function parameters applied for the cyt c spectrum in the presence of SIN-1 and PCL, in 50 mM phosphate buffer, pH 7.4, for 10 and 20 min.

## References

[1] Milani A., Basirnejad M., Shahbazi S., Bolhassani A. (2017). Carotenoids: Biochemistry, pharmacology and treatment. Br. J. Pharmacol..

[2] Young A.J., Lowe G.M. (2001). Antioxidant and prooxidant properties of carotenoids. Arch. Biochem. Biophys..

[3] Polotow T.G., Poppe S.C., Vardaris C.V., Ganini D., Guariroba M., Mattei R., Mattei R., Hatanaka E., Martins M.F., Bondan E.F. (2015). Redox status and neuro inflammation indexes in cerebellum and motor cortex of wistar rats supplemented with natural sources of omega-3 fatty acids and astaxanthin: Fish Oil, Krill Oil, and Algal Biomass. Mar. Drugs.

[4] Yuan J.P., Peng J., Yin K., Wang J.H. (2011). Potential health-promoting effects of astaxanthin: A high-value carotenoid mostly from microalgae. Mol. Nutr. Food Res..

[5] Guerin M., Huntley M.E., Olaizola M. (2003). Haematococcus astaxanthin: Applications for human health and nutrition. Trends Biotechnol..

[6] Kuroki T., Ikeda S., Okada T., Maoka T., Kitamura A., Sugimoto M., Kume S. (2013). Astaxanthin ameliorates heat stress-induced impairment of blastocyst development in vitro: Astaxanthin colocalization with and action on mitochondria. J. Assist. Reprod. Genet..

[7] Bolin A.P., Macedo R.C., Marin D.P., Barros M.P., Otton R. (2010). Astaxanthin prevents in vitro auto-oxidative injury in human lymphocytes. Cell Biol. Toxicol..

[8] Kavitha K., Kowshik J., Kishore T.K., Baba A.B., Nagini S. (2013). Astaxanthin inhibits NF-κB and Wnt/β-catenin signaling pathways via inactivation of Erk/MAPK and PI3K/Akt to induce intrinsic apoptosis in a hamster model of oral cancer. Biochim. Biophys. Acta.

[9] Kim J.H., Choi W., Lee J.H., Jeon S.J., Choi Y.H., Kim B.W., Chang H.I., Nam S.W. (2009). Astaxanthin inhibits H_2_O_2_-mediated apoptotic cell death in mouse neural progenitor cells via modulation of P38 and MEK signaling pathways. J. Microbiol. Biotechnol..

[10] Jiang X., Wang X. (2004). Cytochrome c-mediated apoptosis. Ann. Rev. Biochem..

[11] Byungki J., Sanghwa H. (2006). Biochemical properties of cytochrome c nitrated by peroxynitrite. Biochimie.

[12] Kawai C., Ferreira J.C., Baptista M.S., Nantes I.L. (2014). Not only oxidation of cardiolipin affects the affinity of cytochrome c for lipid bilayers. J. Phys. Chem..

[13] Sharpe M.A., Cooper C.E. (1998). Reaction of nitric oxide with mitochondrial cytochrome c: A novel mechanism for the formation of nitroxyl anion and peroxide. Biochem. J..

[14] Schonhoff M.C., Gaston B., Mannick J.B. (2003). Nitrosylation of cytochrome c during apoptosis. J. Biol. Chem..

[15] Wolf A.M., Asoh S., Hiranuma H., Ohsawa I., Iio K., Satou A., Ishikura M., Ohta S. (2010). Astaxanthin protects mitochondrial redox state and functional integrity against oxidative stress. J. Nutr. Biochem..

[16] Mano C.M., Barros M.P., Faria P.A., Prieto T., Dyszy F.H., Nascimento O.R., Nantes I.L., Bechara E.J. (2009). Superoxide radical protects liposome-contained cytochrome c against oxidative damage promoted by peroxynitrite and free radicals. Free Radic. Biol. Med..

[17] Rajagopal B.S., Edzuma A.N., Hough M.A., Blundell K.L.I.M., Kagan V.E., Kapralov A.A., Fraser L.A., Butt J.N., Silkstone G.G., Wilson M.T. (2013). The hydrogen-peroxide-induced radical behavior in human cytochrome c–phospholipid complexes: Implications for the enhanced pro-apoptotic activity of the G41S mutant. Biochem. J..

[18] Cassina A.M., Hodara R., Souza J.M., Thomson L., Castro L., Ischiropoulos H., Freeman B.A., Radi R. (2000). Cytochrome c nitration by peroxynitrite. J. Biol. Chem..

[19] Roy B., Guha P., Bhattarai R., Nahak P., Karmakar G., Chettri P., Panda A.K. (2016). Influence of lipid composition, pH, and temperature on physicochemical properties of liposomes with curcumin as model drug. J. Oleo Sci..

[20] Pan J., Heberle F.A., Tristram-Nagle S., Szymanski M., Koepfinger M., Katsaras J., Nučerka N. (2012). Molecular structures of fluid phase phosphatidylglycerol bilayers as determined by small angle neutron and X-ray scattering. Biochim. Biophys. Acta.

[21] Xia S., Tan C., Zhang Y., Abbas S., Feng B., Zhang X., Qin F. (2015). Modulating effect of lipid bilayer–carotenoid interactions on the property of liposome encapsulation. Colloids Surf. B Biointerfaces.

[22] Porcelli A.M., Ghelli A., Zanna C., Pinton P., Rizzuto R., Rugolo M. (2005). pH difference across the outer mitochondrial membrane measured with a green fluorescent protein mutant. Biochem. Biophys. Res. Commun..

[23] Matsuyama S., Llopi J., Deveraux Q., Tsien R., Reed J. (2000). Changes in intramitochondrial and cytosolic pH: Early events that modulate caspase activation during apoptosis. Nat. Cell Biol..

[24] Matsuyama S., Reed J.C. (2000). Mitochondria-dependent apoptosis and cellular pH regulation. Cell Death Differ..

[25] Moncelli M.R., Becucci L., Guidelli R. (1994). The intrinsic pKa values for phosphatidylcholine, phosphatidylethanolamine, and phosphatidylserine in monolayers deposited on mercury electrodes. Biophys. J..

[26] Fiuza B., Subelzú N., Calcerrada P., Straliotto M.R., Piacenza L., Cassina A., Rocha J.B., Radi R., de Bem A.F., Peluffo G. (2015). Impact of SIN-1-derived peroxynitrite flux on endothelial cell redox homeostasis and bioenergetics: Protective role of diphenyl diselenide via induction of peroxiredoxins. Free Radic. Res..

[27] Schafer F.Q., Buettner G.R. (2000). Acidic pH amplifies iron-mediated lipid peroxidation in cells. Free Radic. Biol. Med..

[28] Goto S., Kogure K., Abe K., Kimata Y., Kitahama K., Yamashita E., Terada H. (2001). Efficient radical trapping at the surface and inside the phospholipid membrane is responsible for highly potent antiperoxidative activity of the carotenoid astaxanthin. Biochim. Biophys. Acta.

[29] El-Agamey A., Edge R., Navaratnam S., Land E.J., Truscott T.G. (2006). Carotenoid radical anions and their protonated derivatives. Org. Lett..

[30] Liu X., Osawa T. (2002). Cis astaxanthin and especially 9-cis astaxanthin exhibits a higher antioxidant activity in vitro compared to the all-trans isomer. Biochem. Biophys. Res. Commun..

[31] Mortensen A., Skibsted L.H. (2000). Kinetics and mechanism of the primary steps of degradation of carotenoids by acid in homogeneous solution. J. Agric. Food Chem..

[32] Begum S., Cianci M., Durbeej B., Falklöf O., Hädener A., Helliwell J.R., Helliwell M., Regan A.C., Watt C.l.F. (2015). On the origin and variation of colors in lobster carapace. Phys. Chem. Chem. Phys..

[33] Nantes I.L., Faljoni-Alário A., Nascimento O.R., Bandy B., Gatti R., Bechara E.J.H. (2000). Modifications in heme iron of free and vesicle bound cytochrome c by tert-butyl hydroperoxide: A magnetic circular dichroism and electron paramagnetic resonance investigation. Free Radic. Biol. Med..

[34] Fraga C.G., Leibovitz B.E., Tappel A.L. (1988). Lipid peroxidation measured as thiobarbituric acid-reactive substances in tissue slices: Characterization and comparison with homogenates and microsomes. Free Radic. Biol. Med..

[35] Domijan A.M., Ralić J., Radić-Brkanac S., Rumora L., Žanić-Grubišić T. (2015). Quantification of malondialdehyde by HPLC-FL—Application to various biological samples. Biomed. Chromatogr. BMC.

