# Supplementary material for: Astaxanthin Restrains Nitrative-Oxidative Peroxidation in Mitochondrial-Mimetic Liposomes: A Pre-Apoptosis Model
Source: Mar Drugs. 2018 Apr 12;16(4):126. doi: 10.3390/md16040126 (PMC5923413; doi:10.3390/md16040126)
Supplement: Supplementary file 1 [file marinedrugs-16-00126-s001.pdf]

Supplementary Material Figure S1

(A)

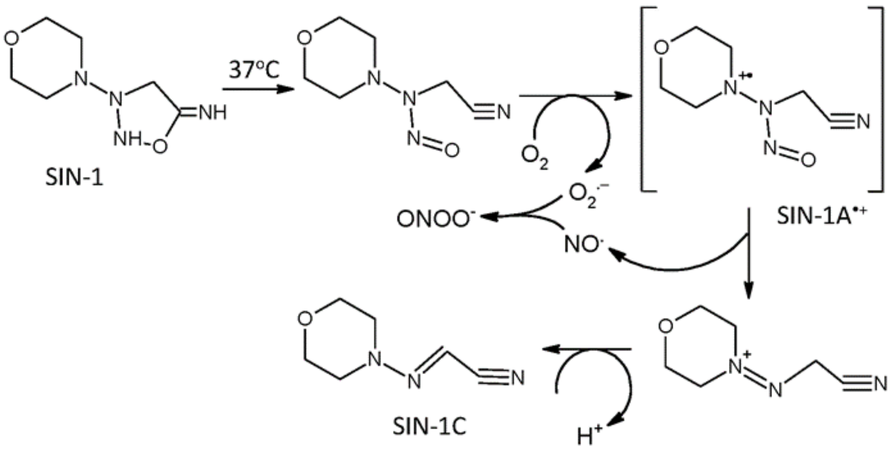

(B)

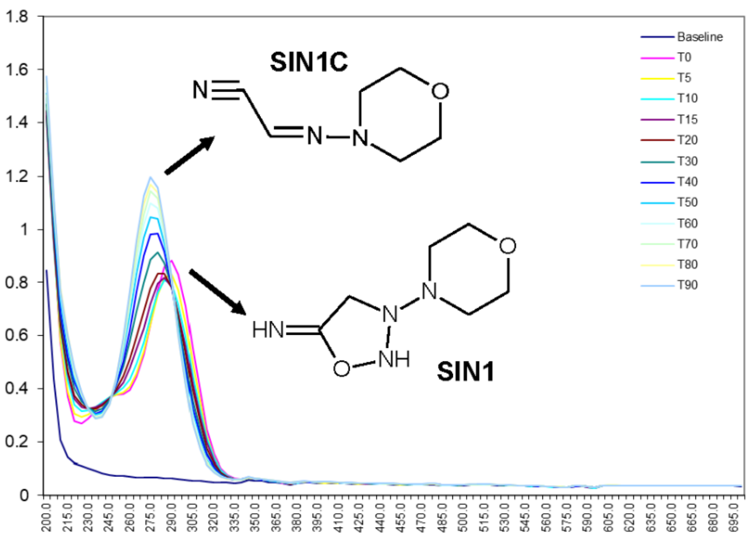

Supplementary Material Figure S2

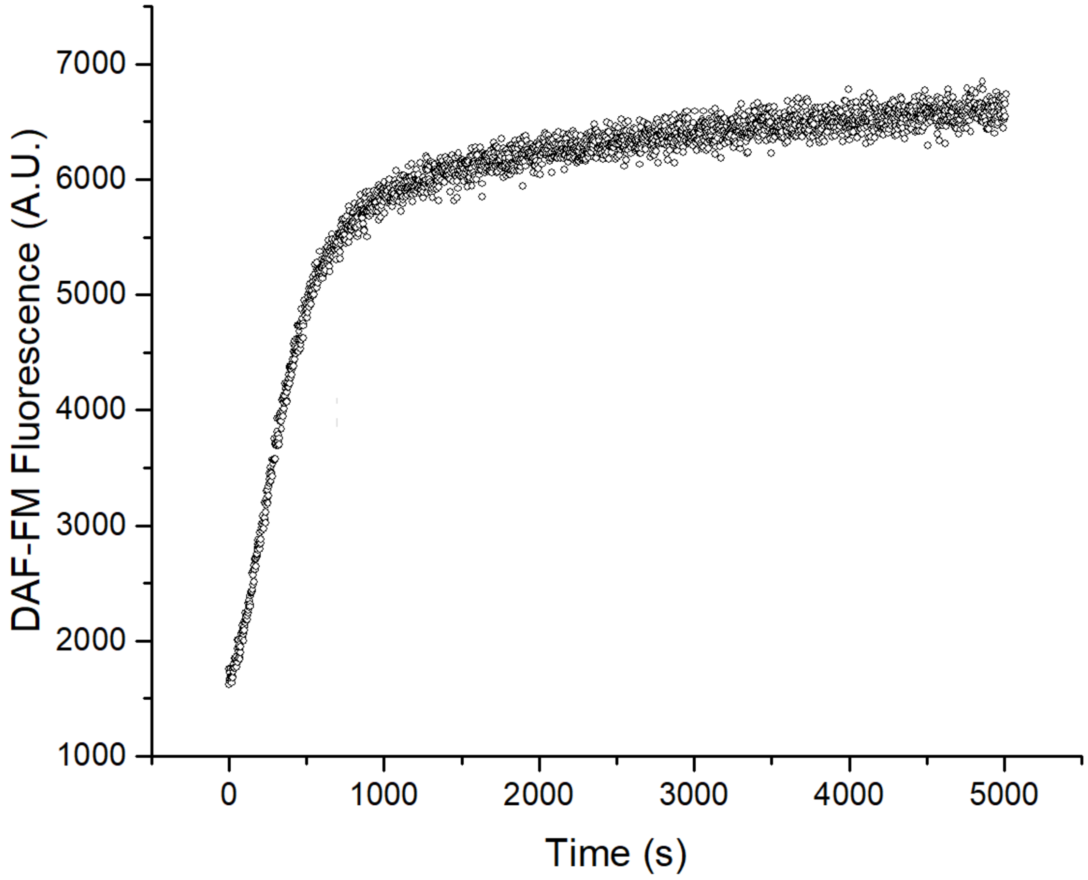

Supplementary Material Figure S3

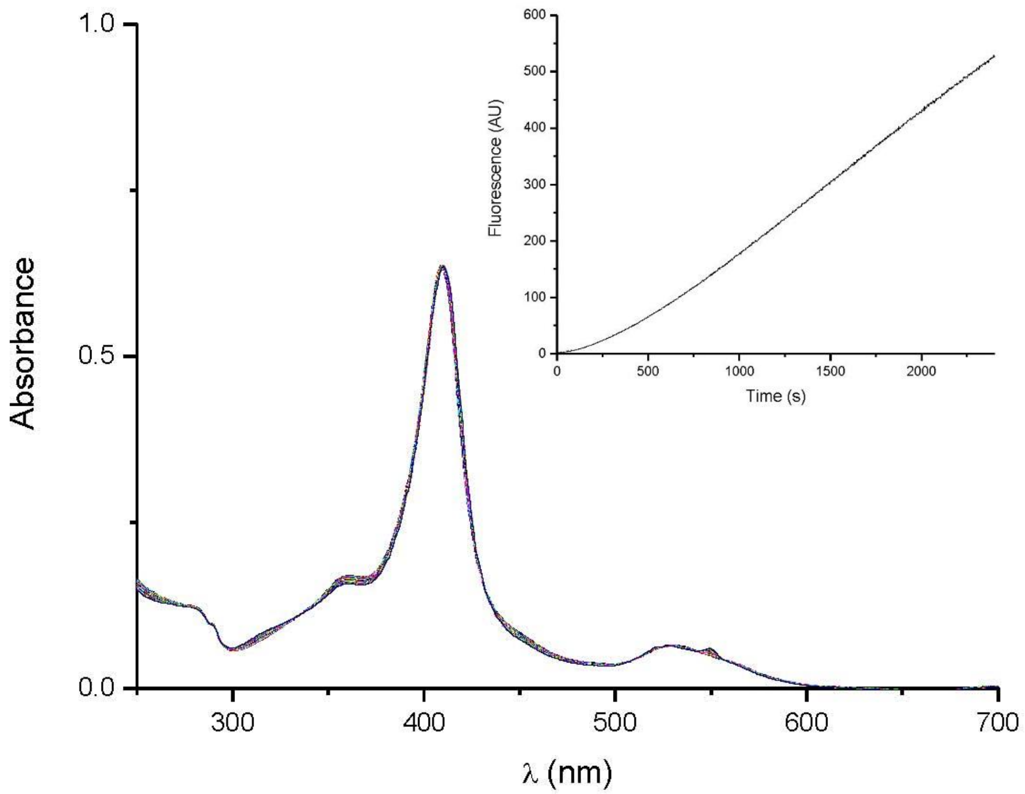

Supplementary Material Table S1

Supplementary Material 3 (Table S3) – Gaussian function parameters applied for the cyt c spectrum in the presence of SIN-1 and PCL, in 50 mM phosphate buffer, pH 7.4, for 10 and 20 min.

System	Time (min)	A	w	y_0	R^2	$(x_c)\lambda_{max}$
Cyt c 50 µM	0	5.9201	14.515	0.2948	0.9884	408.37
	10	5.5736	15.064	0.2647	0.9901	408.30
	20	5.6315	14.853	0.2773	0.9906	408.48
Cyt c 5 µM + SIN-1 50µM	0	4.1607	20.178	0.3382	0.9990	410.53
	10	3.4547	18.200	0.3269	0.9989	409.53
	20	3.5272	18.377	0.3271	0.9990	409.68
Cyt c 50 µM + SIN-1 50µM	0	5.5823	14.948	0.2691	0.9903	408.34
	10	4.8651	15.295	0.2134	0.9898	408.09
	20	5.0023	15.542	0.2080	0.9898	407.95
Cyt c 50 µM + SIN-1 100µM	0	3.8930	14.634	0.2264	0.9912	408.56
	10	4.6444	15.389	0.1993	0.9896	408.00
	20	4.6383	15.335	0.0000	0.9894	408.05

Where: A, area under curve; w, band width (data distribution); y0, curve cut off (baseline); R^2, correlation index; xc, curve peak (calculated λmax).
